# The Impact of Disease-Specific Care Certification on Total Medical Costs for Joint Replacement Surgeries

**DOI:** 10.3390/healthcare13182345

**Published:** 2025-09-18

**Authors:** Yen-Liang Lai, Liang-Hsi Kung, Chih-Ming Kung, Yu-Hua Yan

**Affiliations:** 1 Tainan Municipal Hospital (Managed by Show Chwan Medical Care Corporation), Tainan City 701, Taiwan; lai.larry@msa.hinet.net (Y.-L.L.); 2u0635@mail.tmh.org.tw (L.-H.K.); 2 Department of Nursing, College of Medicine, National Cheng Kung University, Tainan City 701, Taiwan; 3 Department of Information Technology and Communication, Shih Chien University Kaohsiung, Kaohsiung 845, Taiwan; alex@cvig.org

**Keywords:** Disease-Specific Care Certification (DSCC), joint replacement, total medical costs, DRG, healthcare quality, Taiwan, accreditation, resource allocation

## Abstract

**Background/Objectives:** This study investigates the impact of Disease-Specific Care Certification (DSCC) on total medical costs associated with joint replacement surgeries in Taiwan. **Methods:** Using retrospective inpatient data from a regional hospital, we analyzed 660 cases of primary total knee replacement (DRG20903), total hip replacement (DRG20904), and unicompartmental knee replacement (DRG20905) classified under Taiwan’s Tw-DRG system. The dataset covered a 24-month period before certification and a 17-month period after certification, allowing for a comparison of cost changes associated with DSCC implementation. **Results:** While total medical costs increased slightly following certification, the differences across DRG categories were not statistically significant. However, significant increases were observed in rehabilitation costs (all DRGs), surgical costs (DRG20904 and DRG20905), anesthesia costs (DRG20904), and injection-related costs (DRG20905), indicating increased investment in standardized postoperative care. In contrast, blood transfusion and special materials costs significantly decreased in DRG20905, possibly reflecting improved care coordination and resource optimization. Additionally, the proportion of patients with prolonged hospital stays (≥11 days) declined significantly, suggesting potential efficiency gains. **Conclusions:** These findings imply that DSCC may facilitate better resource allocation and clinical standardization without substantially increasing overall medical expenditures, offering valuable insights for hospital administrators and policymakers operating under global budgeting systems.

## 1. Introduction

With global population aging and the rising prevalence of degenerative joint diseases, hip and knee replacement surgeries have become among the most frequently performed orthopedic procedures worldwide. These procedures encompass preoperative evaluation, inpatient treatment, postoperative rehabilitation, and long-term follow-up, placing substantial demands on healthcare resources and hospital budgets. Ensuring patient safety and maintaining care quality—while simultaneously controlling costs and improving efficiency—remains a central challenge for healthcare systems and hospital administrators [1].

To promote standardization and enhance clinical quality, the Taiwan Joint Commission on Hospital Accreditation (JCT) introduced the Disease-Specific Care Certification (DSCC) program in the early 2000s, which was adapted from the U.S. Joint Commission model but localized to Taiwan’s healthcare context. The certification covers a variety of conditions, including cardiovascular disease, stroke, diabetes, infectious diseases, and orthopedic procedures such as total hip and total knee replacements [2,3]. The program emphasizes adherence to clinical practice guidelines, performance measurement, interdisciplinary collaboration, and continuous quality improvement to reduce care variation and improve both patient outcomes and system efficiency [4].

Although previous research suggests that DSCC may contribute to improved care quality, its impact on clinical outcomes and healthcare resource utilization remains inconclusive. Recent reviews and empirical studies in the past decade provide mixed evidence, with some confirming reduced length of stay and costs, while others report no significant improvements in readmissions or complications [5,6]. A systematic review of U.S.-based studies published between 2003 and 2015 identified 15 articles reporting positive associations between certification and care quality. However, only six of these provided empirical data, and most lacked robust statistical models to control for confounding factors [7]. Consequently, the actual effect of DSCC on outcomes such as mortality, readmission rates, and patient satisfaction remains unclear. These findings highlight the need for more rigorous empirical studies—particularly in relation to total medical costs and resource utilization.

From an implementation standpoint, several case studies have demonstrated the pivotal role of Clinical Nurse Specialists (CNSs) in DSCC programs. In one hospital, CNSs led efforts in standardization, team coordination, and performance improvement during the certification process for five service areas, including total hip and total knee replacements [8]. Similarly, in a geriatric hip fracture certification program, CNSs were instrumental in identifying process gaps, developing performance indicators, and leading staff training initiatives—providing valuable insights applicable to orthopedic certifications [9].

In addition, increasing attention has been paid to the relationship between certification programs and healthcare efficiency or cost control. One study reported that DSCC implementation in a U.S. joint replacement center led to a reduction in average length of stay by approximately 0.5 days through coordinated supply management, patient education, risk stratification, and initiation of same-day postoperative physical therapy, without increasing complication rates or reducing patient satisfaction [10]. Another large-scale multicenter study involving 36,935 hip and knee replacements in Denmark showed that enhanced recovery after surgery (ERAS) protocols reduced median hospital stay from three days to one to two days, lowered 90-day readmission rates from 8.6% to 7.7%, and decreased complication rates, illustrating the effectiveness of standardized care pathways [1].

Beyond single-institution and multicenter findings, policy- and payment-related studies have provided indirect evidence of DSCC’s potential to influence cost and efficiency. An analysis based on data from the Michigan Arthroplasty Registry Collaborative Quality Initiative (MARCQI) associated certification with more consistent care, improved patient satisfaction, shorter hospital stays, and reduced costs [11]. Another study evaluating the U.S. Bundled Payments for Care Improvement (BPCI) initiative found average savings of approximately USD 444 per joint replacement episode, primarily due to reduced use of post-acute rehabilitation facilities [6,12,13]. Although hospital stays were slightly shortened, readmission rates remained unaffected. Similarly, a Centers for Medicare & Medicaid Services (CMS) evaluation of the Comprehensive Care for Joint Replacement (CJR) model reported average savings of USD 1012 per procedure (approximately 3.5%), primarily through improved postoperative care coordination [13]. While these studies did not directly evaluate DSCC, they underscore the broader potential of care standardization initiatives to reduce total medical costs and improve system efficiency.

In summary, DSCC and related quality improvement programs promote process standardization, interdisciplinary collaboration, and potentially enhanced cost efficiency. However, studies in Asian healthcare systems remain scarce, and Taiwan in particular lacks empirical analyses of DSCC’s impact under its global budgeting framework [14,15]. This study aims to address this gap by evaluating the influence of DSCC implementation on medical expenditures related to hip and knee replacements using real-world data from a regional hospital in Taiwan. The findings aim to inform healthcare administrators and policymakers operating under global budgeting frameworks.

## 2. Materials and Methods

### 2.1. Study Design

This retrospective observational study examined the impact of Disease-Specific Care Certification (DSCC) on total medical costs and healthcare resource utilization among patients undergoing joint replacement surgery. The study was conducted at a regional hospital in Taiwan, which formally implemented the DSCC program for joint replacement in February 2024. To enhance methodological transparency, the study design is clarified as a pre–post comparison without a concurrent control group, focusing on inpatient cases only. Data were collected over a 41-month period, including 24 months prior to certification (January 2022 to December 2023) and 17 months post-certification (February 2024 to June 2025). Comparative analyses were conducted between the pre- and post-certification periods.

### 2.2. Sample Selection and Exclusion Criteria

The study population consisted of inpatients who underwent joint replacement procedures, classified under Taiwan’s Diagnosis-Related Group (Tw-DRG) system. Specifically, patients were categorized into DRG20903 (primary total knee replacement), DRG20904 (primary total hip replacement), and DRG20905 (unicompartmental knee replacement). Patients who underwent revision surgeries—such as revision of hip or knee replacements, with or without complications or comorbidities—were excluded from the analysis. Exclusion was based on the heterogeneity and higher complexity of revision procedures, which could confound cost analysis given the relatively small sample size.

### 2.3. Data Collection and Analysis

Cost-to-charge ratio (CCR) adjustments were not applied; instead, medical expenditures were determined using the National Health Insurance (NHI) reimbursement schedule to ensure consistency and comparability. All reimbursable inpatient service items were included in the analysis, such as consultation fees, ward charges, dietary services, laboratory tests, radiology, surgical procedures, rehabilitation, anesthesia, blood transfusion, special materials, medication, pharmaceutical services, and injection-related technical fees. Demographic variables (sex, age), clinical characteristics (procedure type), and length of hospital stay were also collected to describe patient characteristics.

A total of 660 inpatient cases were included in the final analysis. After data cleaning and integration using Microsoft Excel(Microsoft Corp., Redmond, WA, USA), statistical analysis was performed with SPSS version 19.0 for Windows, Version 19.0 (IBM Corp., Armonk, NY, USA). Descriptive statistics were used to summarize patient characteristics. Chi-square tests were applied to assess differences in categorical variables, and paired *t*-tests were used to compare medical expenditures before and after DSCC implementation. Independent-sample *t*-tests were additionally used for subgroup comparisons (e.g., sex-based differences).

## 3. Results

### 3.1. Patient Characteristics

A total of 660 inpatient cases were included in the analysis, with comparisons made across certification periods and DRG classifications. Of these, 370 patients received treatment during the pre-certification period and 290 during the post-certification period. The distributions of sex, age, and DRG classifications were comparable between the two groups, with no statistically significant differences observed (*p* > 0.05). However, the proportion of patients with a length of stay ≥11 days significantly decreased following DSCC implementation (*p* = 0.011), suggesting improved efficiency in postoperative management and discharge planning. (Table 1).

### 3.2. DRG20903: Total Knee Replacement

For DRG20903 (total knee replacement), total medical costs increased after certification, although the change was not statistically significant. Among individual cost components, rehabilitation costs showed a significant increase (*p* < 0.001), reflecting intensified use of standardized postoperative rehabilitation protocols. Other categories, including surgical costs, did not demonstrate statistically significant differences (Table 2).

### 3.3. DRG20904: Total Hip Replacement

In DRG20904 (total hip replacement), surgical costs (*p* < 0.001), rehabilitation costs (*p* < 0.05), blood transfusion costs (*p* < 0.05), and anesthesia costs (*p* < 0.05) all increased significantly post-certification. Despite these increases in specific categories, the overall increase in total medical costs for this group remained statistically non-significant (*p* > 0.05), suggesting that DSCC implementation primarily influenced selected care processes rather than overall expenditure (Table 3).

### 3.4. DRG20905: Unicompartmental Knee Replacement

For DRG20905 (unicompartmental knee replacement), significant increases were observed in surgical costs (*p* < 0.001), rehabilitation costs (*p* < 0.001), and injection-related costs (*p* < 0.05). Conversely, blood transfusion costs significantly decreased (*p* < 0.001). Notably, special materials costs also showed a significant reduction post-certification (*p* < 0.05), which may reflect improved cost control or more efficient procurement practices (Table 4).

## 4. Discussion

This study examined the impact of Disease-Specific Care Certification (DSCC) on total medical costs associated with joint replacement surgeries in a regional hospital in Taiwan. Although overall costs showed a slight increase across DRG20903–20905 following DSCC implementation, these differences were not statistically significant. Several specific cost components—particularly rehabilitation, surgical (in DRG20904 and DRG20905), anesthesia (in DRG20904), and injection-related services (in DRG20905)—did show significant increases, indicating that DSCC may influence resource allocation toward standardized, protocol-driven care pathways. Notably, special materials costs significantly decreased in DRG20905, potentially reflecting improved procurement processes or clinical efficiency.

These findings align partially with prior research. A systematic review of DSCC-related studies reported generally positive associations between certification and care quality, although only a minority of studies included empirical data or robust cost analysis [5]. More recent evaluations also suggest mixed results: while some confirm reduced length of stay and lower post-acute costs, others show no significant changes in readmissions or complications [5,6]. This study helps address that gap by offering comparative cost data before and after certification. Similarly, data from the Michigan Arthroplasty Registry Collaborative Quality Initiative suggested greater care consistency and patient satisfaction under DSCC, despite inconclusive findings regarding cost savings [11]. Our results echo this pattern—indicating quality improvements and resource intensification without significant increases in total medical costs.

In terms of efficiency, our findings support previous evidence that standardized recovery protocols can reduce length of stay. The observed increase in rehabilitation costs across all DRGs, coupled with increased surgical costs in DRG20904 and DRG20905, may reflect the adoption of more intensive perioperative protocols post-certification [4]. These results are consistent with ERAS literature showing improved efficiency under standardized pathways [13,15].

Unlike studies linking accreditation to improved mortality outcomes [16,17], this study did not examine clinical outcomes such as complication rates or mortality. However, the significant reduction in blood transfusion costs in DRG20905 may reflect improved perioperative management, indirectly contributing to patient safety benefits.

From a payment system perspective, our findings contrast with bundled payment models such as the Bundled Payments for Care Improvement (BPCI) initiative [12] and the Comprehensive Care for Joint Replacement (CJR) model [13], both of which demonstrated measurable cost savings. Taiwan’s National Health Insurance (NHI) system, which operates under global budgeting and point-based reimbursement, may limit financial incentives for direct cost reductions. Nevertheless, DSCC may still yield value by improving care coordination and standardization, offering efficiency gains without substantial increases in cost, a finding distinct from U.S. bundled payment models [13,18].

Recent studies have further highlighted the importance of care delivery models and surgical techniques in cost control. One study comparing outpatient and inpatient total knee arthroplasty found that outpatient protocols reduced episode-of-care costs by approximately 15%, largely due to decreased inpatient resource utilization [19]. While our study did not assess outpatient care, the observed reduction in prolonged hospital stays aligns with this global shift toward early discharge and cost-effective models. Other evidence indicates that surgical decision-making, such as patellar resurfacing versus selective resurfacing, can influence both outcomes and cost-effectiveness, emphasizing the need for continuous refinement of DSCC pathways [20,21].

This study has several limitations. It was conducted at a single regional hospital, which may limit generalizability. Patient-level clinical characteristics such as comorbidities and surgical complexity were not controlled. In addition, the use of NHI reimbursement data without cost-to-charge conversion may introduce some distortion in cost interpretation. Finally, the 17-month post-certification observation period may be insufficient to capture long-term economic or clinical impacts.

Future studies should adopt multicenter designs, incorporate risk adjustment models, and evaluate quality-of-care outcomes to better assess the long-term value of DSCC under global budgeting systems. As healthcare systems continue to emphasize efficiency, value, and quality, certification programs such as DSCC may play a critical role in promoting standardized, patient-centered care without disproportionately increasing total medical costs.

In Taiwan, the single-payer NHI system provides a unique context for implementing certification programs, promoting consistent cost and quality tracking under centralized financing. Comparable data infrastructure is rare in Asia, but countries like Japan have advanced in this area—its national joint replacement registry, maintained by the Japanese Orthopaedic Association, offers transparent monitoring of surgical trends [22]. A recent region wide review of orthopedic registries confirms the growing effort in Korea, Japan, and Australia, though Taiwan still lacks a central registry [23]. In parallel, Korea’s hospital accreditation program has demonstrated measurable improvements in 30 day mortality among acute myocardial infarction and stroke patients [24].

These examples highlight Taiwan’s potential advantage: under a single-payer, globally budgeted system, rolling out DSCC can yield actionable insights at scale. While Japan leverages registries and Korea validates accreditation through clinical outcomes, Taiwan’s streamlined reimbursement architecture may accelerate implementation and evaluation of certification’s cost-uniform effects within Asian health systems.

## 5. Conclusions

This study evaluated the impact of Disease-Specific Care Certification (DSCC) on total medical costs associated with total and partial joint replacement surgeries in a regional hospital in Taiwan. Although DSCC implementation did not result in a statistically significant increase in overall costs, specific cost components—notably rehabilitation and surgical services in DRG20904 and DRG20905—showed significant increases. These findings suggest that DSCC may promote the reallocation of resources toward standardized, higher-quality care without substantially increasing total medical expenditures.

The observed reduction in prolonged hospital stays further indicates potential efficiency gains. While direct cost savings were not evident under Taiwan’s global budgeting system, targeted investments in key clinical areas may yield long-term value through improved outcomes and reduced post-discharge utilization.

From a clinical and managerial standpoint, DSCC appears to enhance care coordination, adherence to evidence-based pathways, and continuous quality improvement. Healthcare institutions may leverage certification not only as a tool for performance benchmarking but also as a framework for optimizing surgical care delivery and resource use. Future research should assess patient-centered outcomes, including functional recovery and satisfaction, to complement cost analyses and better capture DSCC’s overall value.

At the policy level, these results underscore the importance of aligning certification efforts with reimbursement mechanisms. Although the current point-based payment system may limit financial incentives for quality improvement, supplemental strategies—such as bundled payment pilots or value-based reimbursement models—could enhance the effectiveness and sustainability of DSCC. Expanding the scope of certification and integrating it with broader payment reforms may further strengthen its impact on healthcare quality and efficiency. In the long term, integrating certification with broader payment reforms and regional or national quality registries could strengthen the monitoring, benchmarking, and sustainability of standardized orthopedic care in Taiwan and other single-payer systems.

## 6. Limitations and Future Directions

This study has several limitations that warrant consideration. First, the analysis was conducted at a single regional hospital in Taiwan, which may limit the generalizability of the findings to other healthcare settings. Second, the study relied solely on administrative claims data from the National Health Insurance (NHI) system without access to patient-level clinical information, such as comorbidities, surgical complexity, or postoperative outcomes. Third, cost estimations were based on reimbursement amounts without cost-to-charge ratio (CCR) conversion, which may not fully reflect actual hospital expenditures. Fourth, the relatively short 17-month post-certification observation period may not be sufficient to capture long-term effects, including survival, functional recovery, or sustained cost impacts.

Future research should adopt multicenter designs to enhance external validity and incorporate detailed clinical data to adjust for risk factors and assess quality-of-care outcomes. Further studies could also explore outpatient and post-discharge cost components, patient-reported outcomes, and cost-effectiveness across various payment models. Longitudinal follow-up extending beyond two to three years would allow for the assessment of whether DSCC benefits persist over time or diminish after initial implementation. Evaluating DSCC within the context of Taiwan’s evolving reimbursement policies—such as bundled payments or value-based care initiatives—may provide valuable insights for aligning certification programs with sustainable healthcare financing. Comparative studies between Taiwan and other single-payer or mixed-payment systems could further clarify the generalizability and policy implications of our findings.

In particular, Taiwan’s single-payer global budget system provides a unique natural laboratory for evaluating certification programs. Unlike fragmented multi-payer systems, the uniform reimbursement structure and centralized data allow for more consistent monitoring of costs and outcomes, offering insights that are highly relevant for other Asian healthcare systems seeking to balance efficiency and equity.

## Figures and Tables

**Table 1:** Demographic characteristics of patients before and after DSCC implementation (*n* = 660).

Variable	Category	Pre-Certification (*n* = 370)	Percentage	Post-Certification (*n* = 290)	Percentage	Mean	χ^2^
DRG Classification	20,903	48	7.3	45	6.8		0.257
	20,904	188	28.5	129	19.5		
	20,905	134	20.3	116	17.6		
Sex	Male	113	17.1	97	14.7		0.238
	Female	257	38.9	193	29.2		
Age	<60	36	5.5	29	4.4	72.5	0.993
	60–69	93	14.1	70	10.6		
	70–79	141	21.4	112	17.0		
	≥80	100	15.2	79	12.0		
Length of Stay	<6	186	28.2	178	27.0	5.6	0.011
	6–7	141	21.4	86	13.0		
	8–10	28	4.2	22	3.3		
	≥11	15	2.3	4	0.6		

Note: Data period: January 2022 to June 2025. Chi-square test used for categorical variables. *p*-value shown for between-group differences.

**Table 2:** Comparison of medical costs for DRG20903 (total knee replacement) before and after DSCC implementation (*n* = 93).

Category	Pre-Certification (*n* = 48)		Post-Certification (*n* = 45)		t-Value
	Mean	±SD	Mean	±SD	
Consultation fees	3300	1700	3385	1114	−0.282
Ward fees	6754	6125	6509	3733	0.231
Dietary and feeding fees	8	56	17	119	−0.504
Lab fees	1791	1039	1469	842	1.636
Radiology Fee	1130	696	4196	998	0.677
Treatment and Procedure Fees	1053	1614	1099	1440	−0.143
Surgery Fees	30,743	5088	33,054	6167	−1.976
Rehabilitation Fees	118	333	1378	1316	−6.417 ***
Blood Transfusion Fees	2540	4927	1758	3432	0.882
Dialysis Fees	0	0	0	0	0
Anesthesia Fees	6421	1844	6199	1722	0.600
Special Materials Fees	46,173	13,190	52,066	23,782	−1.490
Medication Fees	1196	1075	1124	463	0.416
Pharmacist Service Fees	651	215	646	157	0.126
Injection Fees	137	91	151	60	−0.870
Total Medical Costs	101,604	34,575	117,491	49,810	−1.796

Note: All values are presented as mean ± standard deviation. *** *p* < 0.001. All costs are expressed in New Taiwan Dollars (TWD). DSCC = Disease-Specific Care Certification.

**Table 3:** Comparison of medical costs for DRG20904 (total hip replacement) before and after DSCC implementation (*n* = 317).

Category	Pre-Certification (*n* = 188)		Post-Certification (*n* = 129)		t-Value
	Mean	±SD	Mean	±SD	
Consultation fees	4701	4023	4365	1889	0.884
Ward fees	7834	6750	7789	4757	0.066
Dietary and feeding fees	94	765	11	90	1.227
Lab fees	3267	2188	3354	2406	−0.332
Radiology Fee	1410	1418	1574	1631	−0.949
Treatment and Procedure Fees	2571	4543	1998	1931	1.350
Surgery Fees	17,663	3579	20,071	3983	−5.618 ***
Rehabilitation Fees	146	519	329	819	−2.424 *
Blood Transfusion Fees	1268	2493	685	1602	2.342 *
Dialysis Fees	305	1822	317	2088	−0.057
Anesthesia Fees	5516	1245	5900	1413	−2.551 *
Special Materials Fees	44,115	12,364	44,120	7929	−0.004
Medication Fees	1712	3093	1586	3196	0.350
Pharmacist Service Fees	661	231	632	237	1.088
Injection Fees	150	74	143	68	0.824
Total Medical Costs	91,270	23,278	93,211	20,477	−0.765

Note: All values are presented as mean ± standard deviation. * *p* < 0.05, *** *p* < 0.001. All costs are expressed in New Taiwan Dollars (TWD). DSCC = Disease-Specific Care Certification.

**Table 4:** Comparison of medical costs for DRG20905 (unicompartmental knee replacement) before and after DSCC implementation (*n* = 250).

Category	Pre-Certification (*n* = 134)		Post-Certification (*n* = 116)		t-Value
	Mean	±SD	Mean	±SD	
Consultation Fees	3499	900	3373	829	1.145
Ward Fees	6218	2379	5821	2042	1.405
Dietary	4	55	3	36	0.235
Laboratory	1720	1324	1768	1207	−0.293
Radiology Fee	723	459	731	574	−0.130
Treatment and Procedure Fees	1068	1227	748	395	2.690 **
Surgery Fees	29,139	4374	31,901	4308	−5.013 ***
Rehabilitation Fees	1163	609	2004	1086	−7.676 ***
Blood Transfusion Fees	774	1557	189	793	3.658 ***
Dialysis Fees	0	0	70	761	−1.075
Anesthesia Fees	6094	1138	5821	878	2.103 *
Special Materials Fees	46,462	7653	44,518	7515	2.020 *
Medication Fees	1293	952	1268	991	0.203
Pharmacist Service Fees	639	153	606	155	1.687
Injection Fees	13	47	135	43	−2.105 *
Total Medical Costs	92,350	16,582	91,666	15,532	0.335

Note: All values are presented as mean ± standard deviation.* *p* < 0.05, ** *p* < 0.01, *** *p* < 0.001. All costs are expressed in New Taiwan Dollars (TWD). DSCC = Disease-Specific Care Certification.

## Data Availability

Data cannot be made publicly available owing to the fact that the privacy of individual participants cannot be compromised. However, the dataset is available from the corresponding author upon reasonable request.
